# The Opportunistic Pathogen *Vibrio vulnificus* Produces Outer Membrane Vesicles in a Spatially Distinct Manner Related to Capsular Polysaccharide

**DOI:** 10.3389/fmicb.2017.02177

**Published:** 2017-11-07

**Authors:** Cheri M. Hampton, Ricardo C. Guerrero-Ferreira, Rachel E. Storms, Jeannette V. Taylor, Hong Yi, Paul A. Gulig, Elizabeth R. Wright

**Affiliations:** ^1^Division of Pediatric Infectious Diseases, Emory University School of Medicine, Children’s Healthcare of Atlanta, Atlanta, GA, United States; ^2^Robert P. Apkarian Integrated Electron Microscopy Core, Emory University, Atlanta, GA, United States; ^3^Department of Molecular Genetics and Microbiology, College of Medicine, University of Florida, Gainesville, FL, United States

**Keywords:** Vibrionaceae, *Vibrio vulnificus*, outer membrane vesicles, cryo-electron microscopy, cryo-electron tomography

## Abstract

*Vibrio vulnificus*, a bacterial species that inhabits brackish waters, is an opportunistic pathogen of humans. *V. vulnificus* infections can cause acute gastroenteritis, invasive septicemia, tissue necrosis, and potentially death. Virulence factors associated with *V. vulnificus* include the capsular polysaccharide (CPS), lipopolysaccharide, flagellum, pili, and outer membrane vesicles (OMVs). The aims of this study were to characterize the morphology of *V. vulnificus* cells and the formation and arrangement of OMVs using cryo-electron microscopy (cryo-EM). cryo-EM and cryo-electron tomography imaging of *V. vulnificus* strains grown in liquid cultures revealed the presence of OMVs (diameters of ∼45 nm for wild-type, ∼30 nm for the unencapsulated mutant, and ∼50 nm for the non-motile mutant) in log-phase growth. Production of OMVs in the stationary growth phase was limited and irregular. The spacing of the OMVs around the wild-type cells was in regular, concentric rings. In wild-type cells and a non-motile mutant, the spacing between the cell envelope and the first ring of OMVs was ∼200 nm; this spacing was maintained between subsequent OMV layers. The size, arrangement, and spacing of OMVs in an unencapsulated mutant was irregular and indicated that the polysaccharide chains of the capsule regulate aspects of OMV production and order. Together, our results revealed the distinctive organization of *V. vulnificus* OMVs that is affected by expression of the CPS.

## Introduction

*Vibrio vulnificus*, a halotrophic Gram-negative bacterium that lives in brackish estuarine waters, is associated with shellfish in natural environments (DePaola et al., 1997) and is an opportunistic pathogen of humans (Jones and Oliver, 2009). It is the major cause of reported deaths from the consumption of raw or undercooked seafood in the United States (Strom and Paranjpye, 2000; Bross et al., 2007). Many decades of research have focused on defining the molecular pathogenesis of *V. vulnificus*. Once the bacteria invade human tissues, they multiply rapidly and cause a number of conditions including acute gastroenteritis, invasive septicemia, tissue necrosis, and potentially death (Jones and Oliver, 2009). A number of studies have identified many of the virulence factors associated with *V. vulnificus* (Gulig et al., 2005). Some of the virulence factors identified include external structures of the bacteria such as the capsular polysaccharide (CPS), lipopolysaccharide (LPS) (Ellis and Kuehn, 2010; Vanaja et al., 2016), flagellum, pili (Srivastava et al., 2009), as well as the more recently recognized process of outer membrane vesicle (OMV) production (Kim et al., 2010; Kulp and Kuehn, 2010; Guerrero-Ferreira et al., 2011). However, little is known about the ultrastructure of *V. vulnificus* or the structure of CPS, flagellum, pili, and OMVs or their relationships to other cellular components.

The *V. vulnificus* capsule is composed of varying types and amounts of CPS. The *V. vulnificus* CPS is considered to be the organism’s most important virulence factor, and it is used primarily to avoid phagocytosis by host defense cells such as neutrophils (Hayat et al., 1993; Simonson and Siebeling, 1993; Wright et al., 1999; Hilton et al., 2006). Its expression has been demonstrated to undergo phase variation (Chatzidaki-Livanis et al., 2006; Hilton et al., 2006). Acapsular strains have been proven to be non-infectious (Wright et al., 1990).

Similarly, the immunogenic LPS, which has been implicated as a *V. vulnificus* pyrogen causing endotoxic shock (McPherson et al., 1991; Jones and Oliver, 2009), extends outward from the outer membrane. It is anchored in the outer membrane by its lipid A component, followed by a core oligosaccharide with the O antigen distal to the membrane. The synthesis of the O antigen may share genetic loci with CPS biosynthesis (Nakhamchik et al., 2007) and, hence, may resemble the CPS (Wright et al., 1999), making it difficult to distinguish between the two polysaccharides antigenically or chemically.

Imaging the bacterial capsule is problematic in two ways. First, the capsule has a high water content, which results in shrinking during the dehydration process of traditional staining (Beveridge and Graham, 1991). Second, the CPS and LPS are largely composed of flexible sugar polymers which have little electron density, resulting in poor image contrast. There is also propensity for these sugars to be damaged during handling and processing protocols. Several groups have developed staining and preservation methods of the bacterial capsule for conventional transmission electron microscopy (TEM) applications (Jacques and Graham, 1989; Wright et al., 1990; Fassel and Edmiston, 1999; Hammerschmidt et al., 2005). Imaging studies of *V. vulnificus* using this methodology have demonstrated the presence or absence of the bacterial capsule in opaque (capsulated) and transparent (non-capsulated) strains (Wright et al., 1990), and semi-quantitative differences during phase transitions (Wright et al., 1999).

**FIGURE 1:**
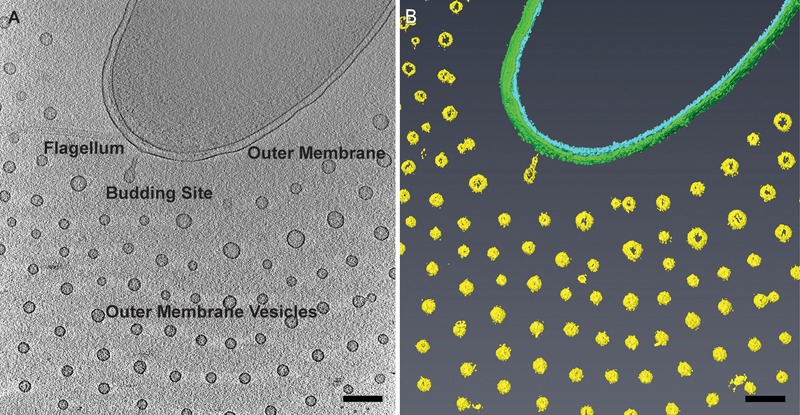
Planar arrangement of *Vibrio vulnificus* outer membrane vesicles (OMVs). **(A)** Cryo-electron tomogram slice of *V. vulnificus* producing outer membrane vesicles. Adapted from Hampton et al. (2015). **(B)** Segmentation of the 3D reconstruction. Inner membrane, cyan; outer membrane, green; OMVs, yellow. Scale bars, 200 nm.

*Vibrio vulnificus*, like many Gram-negative bacteria, produces OMVs that may contribute to pathogenicity. It was recently found that these OMVs can induce host cell death and may carry the cytolysin–hemolysin (VvhA), which induces cytotoxicity (Kim et al., 2010). The production of OMVs by Gram-negative bacteria is an active process that takes place when a section of the outer membrane bulges off to form periplasm-containing, round vesicles without causing bacterial lysis (**Figure 1**) (Kuehn and Kesty, 2005). OMVs directly influence bacterial-host interaction, specifically bacterial pathogenesis, through their ability to modulate immune responses, contribute to biofilm formation, and deliver toxins and other virulence factors to host cells (Kulp and Kuehn, 2010; MacDonald and Kuehn, 2012). Conventional TEM has shown the presence of these outer membrane derived vesicles, particularly for *Pseudomonas aeruginosa* (Beveridge and Graham, 1991; Beveridge, 1999), as well as for *Vibrio cholerae* and *Neisseria meningitidis* serogroup B, which have been used in the production of OMV-based vaccine development (Oster et al., 2005; Schild et al., 2008; Holst et al., 2009; Bishop et al., 2012).

Cryo-electron tomography (cryo-ET) produces native-state three-dimensional (3D) structures of frozen-hydrated cells and their subcellular material at macromolecular resolution (Oikonomou et al., 2016). In the present paper, we use cryo-ET to define the 3D organization of *V. vulnificus* cells grown in Luria Bertani (LB) media. We employed cryo-ET, cryo-preservation techniques combined with freeze substitution, and other conventional electron microscopy methods to systematically examine the relationship between the CPS and OMV release and distribution around the cell. Our examination of capsulated, non-capsulated, and motility-inhibited *V. vulnificus* strains provided a correlation between the presence of the capsule and the regulation and ordered arrangement of OMVs surrounding individual cells. The novel spatial arrangement of the *V. vulnificus* OMVs suggests a unique method for their production and/or purpose.

## Materials and Methods

### Bacterial Strains and Growth Conditions

Bacterial strains used in this study are presented in **Table 1**. *V. vulnificus* strains were grown in LB media consisting of 0.5% (w/v) yeast extract, 1% (w/v) tryptone, and 1% (w/v) NaCl, pH 7.0. Plating medium contained 1.5% (wt/vol) Bacto agar. *V. vulnificus* strains were stored as 20% glycerol stocks at -80°C. Cultures were first streaked on LB plates and incubated overnight at 30°C. Individual colonies were used to inoculate 20 mL LB broth and were grown overnight at 30°C with vigorous shaking (250 rpm). Growth kinetics experiments were performed in three independent experiments with all *V. vulnificus* strains to determine the mid-log growth point for optimal OMV production. Briefly, liquid starter cultures were grown statically overnight, then a 1:2000 dilution of the starter culture was inoculated into fresh media and grown under standard conditions. Optical density (OD_600_) readings were taken from flasks at 30 min intervals for 7–12 h post-inoculation. To simulate growth phase synchronization, 100 μL of overnight culture was inoculated into 100 mL of fresh LB broth and grown to mid-log phase (OD_600_ ≅ 0.5, ∼3–4 h).

**Table 1:** Bacterial strains used in this study.

Species	Strain	Genotype	Description/phenotype	Source
*Vibrio vulnificus*	CMCP6		Wild-type, biotype 1 clinical isolate	1
	FLA1009	CMCP6 *wza*::Tn*PhoA*	Polar Tn*PhoA* mutant/unencapsulated	2
	FLA674	CMCP6 Δ*motAB*::*aph*	Flagellar motor mutant/flagellated, non-motile	2
	FLA810	Δ*flaCDE*::*aph* Δ*flaFBA*::*cat*	Lacks all six flagellin genes, no flagellum, non-motile	2
	FLA757	*flaFBA*::*cat*	*flaCDE* revertant of Δ*flaCDE*::*aph*, Δ*flaFBA*::*cat*/flagellated, motile	3
	FLA674 (pGTR1190)	Δ*motAB*::*aph* (pGTR1190 *motAB*)	Complementation of Δ*motAB*::*aph* with plasmid-encoded *motAB/*Motile	3
*Neisseria meningitidis*	Serogroup B		Wild-type	4

*Strain sources: 1. Clinical isolate, Chonnam National University Hospital (Kim et al., 2003).*

*2. USER friendly cloning and chitin-based transformation (Gulig et al., 2009).*

*3. Gulig lab (unpublished).*

*4. David S. Stephens lab, Emory University.*

*Neisseria meningitidis* B and its CPS mutant, m7, were streaked on GC base agar (Difco) plates supplemented with glucose and iron and grown overnight at 37°C with 5% carbon dioxide. Liquid cultures were aerated in GC broth supplemented with glucose, iron, and 4.3% sodium bicarbonate overnight at 30°C. The following morning the culture was diluted into fresh media and grown to approximately mid-log phase.

### Motility Assays

To determine how specific mutations affected the motility of *V. vulnificus* strains, we carried out motility assays. Briefly, LB plates containing 0.3% (wt/vol) agar were spot-inoculated with 1 μL of culture normalized to OD_600_ = 1 and incubated at 30°C. Motility zones were measured after 4 and 6 h. Cultures were spotted in triplicate, and the experiment was repeated three times. The same was repeated for complemented and reverted strains, however, these were photographed after 18 h due to slower growth.

### Conventional TEM

#### Lysine Acetate-Ruthenium Red

One inoculating loop-full of strain CMCP6 (wild-type) and FLA1009 (*wza*::TnPhoA) was gently re-suspended in a series of 0.1 M cacodylate buffer-based fixatives with a pH range of 7.2–7.4. The first fixative contained 2% paraformaldehyde, 2.5% glutaraldehyde, 0.075% ruthenium red, and 0.075 M lysine acetate. The second contained 2% paraformaldehyde, 2.5% glutaraldehyde, and 0.075% ruthenium red and the third fixative contained 1% osmium tetroxide and 0.075% ruthenium red (Hammerschmidt et al., 2005). Stained cells were then dehydrated through a graded ethanol series and embedded in epoxy resin. 70 nm thick sections were cut and imaged on a JEOL JEM-1400 TEM (JEOL Ltd., Tokyo, Japan) operated at 120 kV.

#### High Pressure Freezing/Self-pressurized Rapid Freezing

Colonies were taken from the surface of LB agar plates grown overnight. For high pressure freezing (HPF), a single colony was transferred to a planchette and frozen in a Bal-Tec HPM 010 high pressure freezer. Planchettes were split, and frozen cells were freeze substituted with 2% OsO4, 0.1% uranyl acetate in acetone, embedded in epoxy resin, and sectioned (McDonald, 2007). For self-pressurized rapid freezing (SPRF), enough PBS buffer (∼10 μL) was added to the colonies to facilitate transfer by suction into the (0.65 mm OD, 0.35 mm ID) tube (approximate volume = 1.54 μL). Both ends of the tube were crimped closed with pliers, and the tube was manually plunged into liquid nitrogen-cooled ethane (-180°C) (Leunissen and Yi, 2009). Frozen tubes were cut open under liquid nitrogen, and the frozen contents extruded and subjected to freeze substitution in a Leica AFS with 2% OsO4, 0.1% uranyl acetate in acetone. Samples were then embedded in epoxy resin, 70 nm thick sections were cut and imaged on a JEOL JEM-1400 TEM (JEOL Ltd., Tokyo, Japan) operated at 120 kV.

### Cryo-Electron Microscopy

The process of OMV release, the structure of discharged OMVs, and the morphology and integrity of *V. vulnificus* cells were characterized by cryo-electron microscopy (cryo-EM) and cryo-ET. Non-centrifuged 4 μL aliquots of bacterial cultures in exponential growth phase were combined with 1 μL of 10 nm gold fiducials (Sigma-Aldrich) and plunge frozen on glow-discharged, 200 mesh, copper Quantifoil grids (Quantifoil, Germany) in liquid ethane using a Vitrobot Mark III system (FEI, Hillsboro, OR, United States). The grids were observed in a JEOL JEM-2200FS field emission TEM (FEG-TEM) (JEOL Ltd., Tokyo, Japan) operating at 200 kV and equipped with an in-column energy filter, a DE-20 direct detection device (Direct Electron LP, San Diego, CA, United States), and a 4k × 4k UltraScan 4000 CCD camera (Gatan, Pleasanton, CA, United States). Single axis tilt series were acquired with SerialEM (Mastronarde, 2005), with an increment of 2° covering -60° to +60°. The cumulative dose was under 150 electrons/Å^2^ and the defocus was between -4 and -10 μm. The tomograms were reconstructed by r-weighted back-projection using IMOD (Kremer et al., 1996; Mastronarde, 1997). Volume-rendered segmentations were performed manually using the Amira package (FEI Visualization Sciences Group, Hillsboro, OR, United States).

### Data Analysis

OMV diameters and distances from the center of each OMV to the outer membrane of the closest *V. vulnificus* cell were measured using IMOD’s drawing tools. Graphs were made in Prism software (GraphPad Software, Inc., La Jolla, CA, United States). Delaunay triangulations to determine distance to nearest neighbor were performed in ImageJ/Fiji (Plugins>Analyze>Delaunay Voronoi) (Schindelin et al., 2012).

## Results

### Morphological Characterization of *V. vulnificus*

**FIGURE 2:**
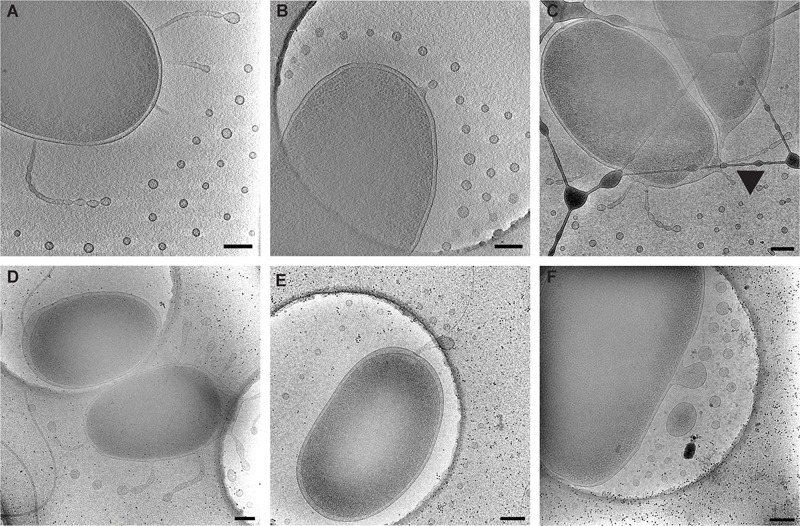
Types of observed vesicle formation in *V. vulnificus*. **(A)** Segmented tubes and individual vesicles. **(B)** Individual outer membrane vesicle budding. **(C)** Segmented tubes seen at the cell division site (arrowhead). **(D)** Loose membranes due to peptidoglycan disruption. **(E)** Shedding of flagellar sheath. **(F)** Membrane blebbing from a membrane disruption. Panels **(A–C)** are typical arrangements for early to mid-log-phase cultures. Panels **(D–F)** are more common for late log-phase and early stationary-phase cultures. Scale bars, 200 nm.

*Vibrio vulnificus* cells were dispersed on the cryo-EM grids as 1.5–3 μm long bacteria. Cell morphology was consistent with Gram-negative species in which the cell cytoplasm was surrounded by the cell wall, which was composed of an inner membrane, peptidoglycan layer, and an outer membrane. Most cells maintained a single, polar membrane-sheathed flagellum, and occasionally pili were observed. OMVs were seen in abundance surrounding the capsulated strains and were arranged in a single plane as concentric rings (**Figures 1, 2A,B**). Also observed were the presence of tubes of outer membrane that pinched off into individual vesicles at the distal tips, forming a “beads-on-a-string” appearance (**Figure 2A**). These tubes were seen at the cell poles as well as at the division plane of dividing cells (**Figure 2C**).

### Mechanism of OMV Release and Relationship to Growth Stage

**FIGURE 3:**
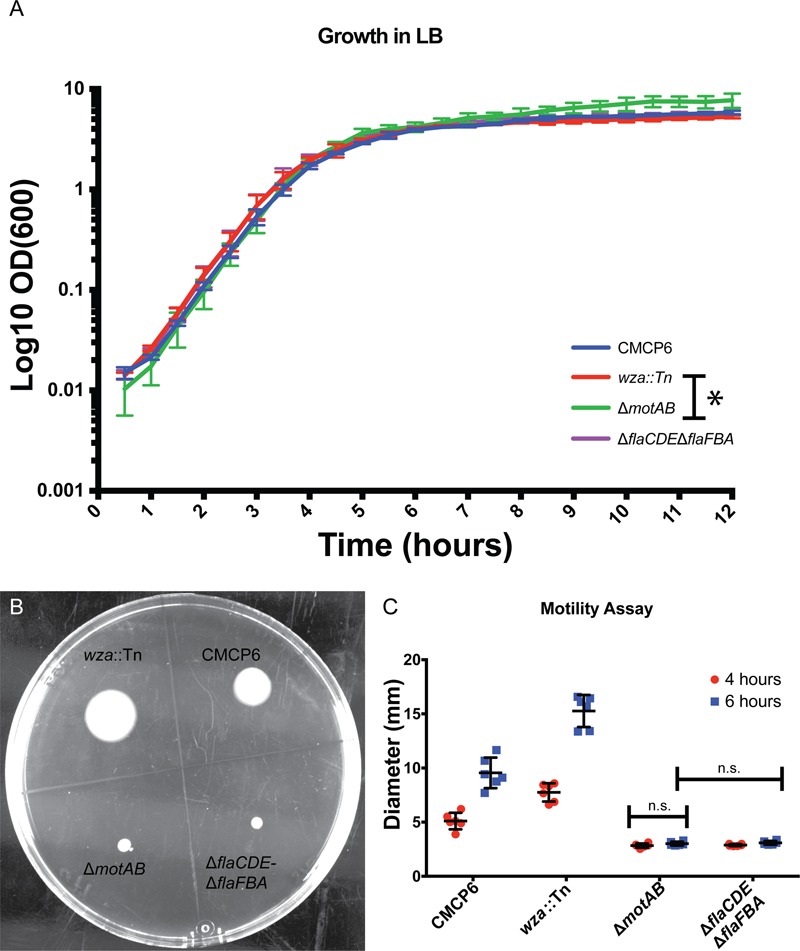
Growth characteristics of *Vibrio vulnificus*. **(A)** *Vibrio vulnificus* growth in LB media. Time points were taken every 30 min for 12 h. Data points are averages from three separate experiments. **(B)** Soft-agar motility assay plate at 6 h post-inoculation. The acapsular mutant had greater motility than the wild-type. The flagellar (*fla*) and motor (*mot*) mutants were non-motile. **(C)** Plot of three separate motility experiments with measurements taken at 4 and 6 h. ANOVA *P*-value <0.0001. Pairwise *t*-tests between all data groups with a *P*-value <0.05 showed significance for all groups except those noted as not significant (n.s.). ^∗^Indicates a statistical difference between the two strains.

To define the growth phases for *V. vulnificus* under our lab conditions, growth curves were performed. Overnight cultures were diluted into fresh LB media, and samples were taken at 30 min intervals for 12 h. A plot of OD_600_ versus time (**Figure 3A**) revealed an approximate mid-log time point of 3.5–4 h and a late-log time point of 7–8 h. No growth defects were observed for the *V. vulnificus* strains examined. This was important to define because basic cell morphology is known to be dependent on growth phase of the cells, whether they are actively dividing or are in stationary phase. In addition, the expression of cell factors such as CPS is reported to peak during log-phase growth and decline during stationary phase (Wright et al., 1999). Differences in basic cell morphology can be seen in **Figure 2. Figures 2A–C** are from cells vitrified during mid-log growth phase. Cells have compact spacing between inner and outer membranes, with tubes and OMVs exiting the membrane via narrow, defined constrictions. **Figures 2D–F** represent morphologies seen at late-log growth phase. There is greater and more irregular spacing between the inner and outer membranes, suggesting a loss of peptidoglycan adhesiveness which results in a less controlled blebbing or sloughing of the outer membrane. **Figure 2E** shows another phenomenon associated with older cultures, which is flagellar sheath shedding to produce vesicles. This is a first reporting of this phenomenon in *V. vulnificus*, but it has been recently proposed that flagellar sheath shedding, although different in appearance, is a principal mechanism for LPS shedding via the outer membrane in *Vibrio fischeri*, and this shedding is attributed to rotation of the flagellum (Aschtgen et al., 2016).

### Quantification of OMVs Produced by *V. vulnificus* Strains

**FIGURE 4:**
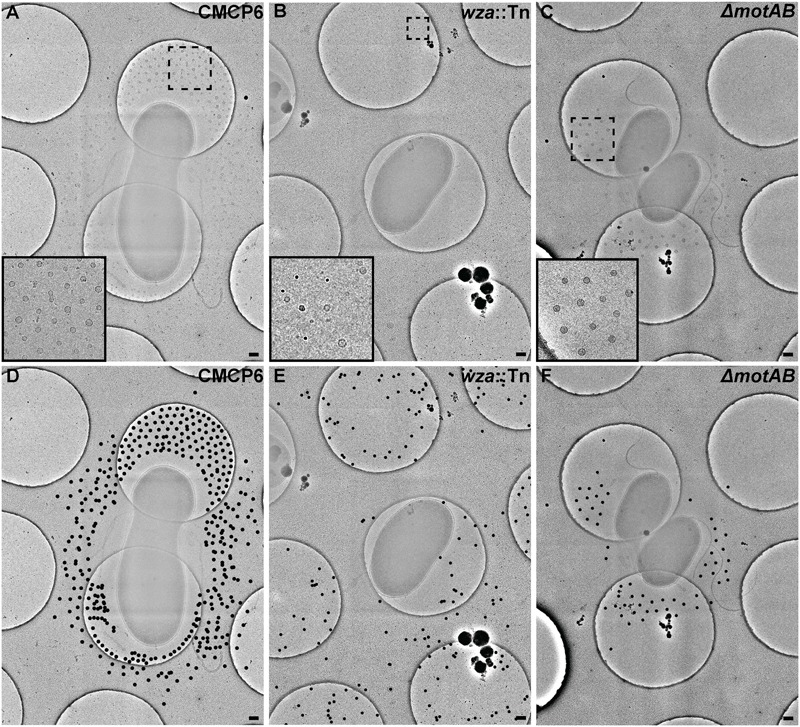
Outer membrane vesicle arrangement and distribution in *Vibrio vulnificus*. **(A,D)** A 3 × 3 image montage of wild-type CMCP6 showing characteristic net-like OMV arrangement around the cell with regular spacing between OMVs and a pronounced empty zone proximal to the outer membrane. Inset in **(A)** is magnified 2.5×. Black dots in **(D)** represent centers of OMVs seen unobstructed in **(A)**. **(B,E)** A 3 × 3 image montage of the *wza*::TnPhoA unencapsulated (FLA1009) mutant showing the more random distribution of OMVs around the cell. Inset in **(B)** is magnified 5×. **(C,F)** A 3 × 3 image montage of Δ*motAB* flagellar motor mutant (FLA674) showing a similar OMV arrangement and distribution as wild-type, but having reduced OMV numbers. Inset in **(C)** is magnified 2.5×. Image montage area = 7.5 μm^2^. Scale bars, 200 nm.

**FIGURE 5:**
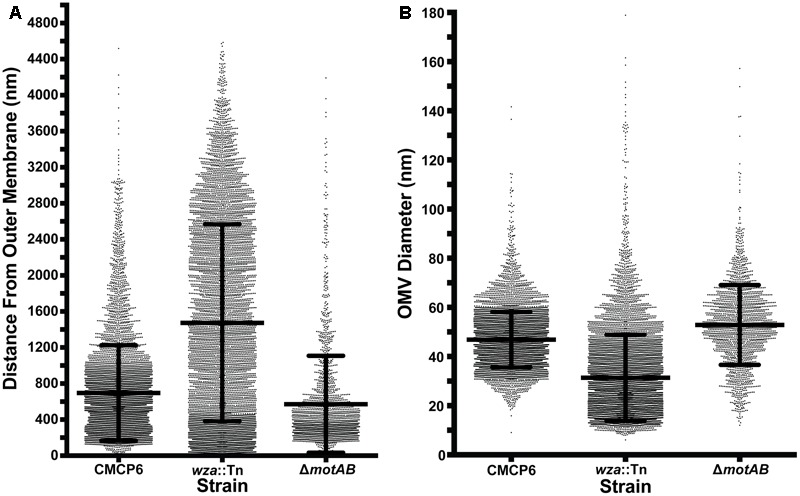
OMV measurement data. **(A)** Distance of OMVs from the outer membrane of the nearest cell. All distances were measured within a 3 × 3 montage area of ∼753 nm. Total number of cells measured: wild-type CMCP6 = 108, *wza*::TnPhoA unencapsulated mutant (FLA1009) = 167, Δ*motAB* flagellar motor mutant (FLA674) = 148. Average number of OMVs per cell: wild-type CMCP6 = 63, *wza*::TnPhoA unencapsulated mutant (FLA1009) = 54, Δ*motAB* flagellar motor mutant (FLA674) = 17. Of note is the relatively small number of OMVs closer than 200 nm to the outer membrane in strains possessing a capsule (wild-type and motor mutant), while the unencapsulated strain has many OMVs at distances less than 200 nm. The overall range of distributions for the capsulated strains is tighter than that of the unencapsulated strain. **(B)** Diameters of OMVs. Diameters for the two capsulated strains are in the 30–70 nm range, while OMVs for the unencapsulated strain are smaller and have a broader range of sizes. Number of OMVs measured: wild-type CMCP6 = 6546, *wza*::TnPhoA unencapsulated mutant (FLA1009) = 7676, Δ*motAB* flagellar motor mutant (FLA674) = 1965.

To avoid complications in OMV quantification due to purification of vesicles and membrane fragments from bulk culture (Kulp and Kuehn, 2010), we relied on quantification of OMVs directly from images of individual cells and their immediate surrounding area. We imaged vitrified *V. vulnificus* cells at mid-log growth by acquiring 3 × 3 image montages at 10,000× magnification to include an area of ∼7.5 μm^2^. The general appearance and OMV distribution for each strain are shown in **Figure 4**. Within this area, all OMVs were counted and their distance from the nearest outer membrane measured. Individual OMV diameters were also recorded. We performed this analysis for three cell strains: wild-type (CMCP6), an acapsular mutant (*wza*::TnPhoA; FLA1009), and a non-motile mutant (Δ*motAB*; FLA674). These measurements are plotted in **Figure 5**. The total number of cells measured was for wild-type CMCP6 = 108, *wza*::TnPhoA acapsular mutant (FLA1009) = 167, Δ*motAB* flagellar motor mutant (FLA674) = 148. The average number of OMVs per cell for wild-type CMCP6 = 63, *wza*::TnPhoA (FLA1009) = 54, Δ*motAB* (FLA674) = 17. For wild-type *V. vulnificus*, the distance from the outer membrane fell largely within a defined ∼1 μm radius (mean = 695 nm). The unencapsulated mutant *wza*::TnPhoA, in distinct contrast, showed an equal number of OMVs but a very broad range of OMV distribution, from just on the outer membrane up to ∼3.5 μm away (mean = 1.5 μm). For the non-motile Δ*motAB* mutant, there were far fewer OMVs per individual cell compared to wild-type or unencapsulated mutants. Their distribution was over a much narrower range than wild-type, only up to ∼1 μm away (mean = 570 nm). Perhaps the most significant observation was the distinct lack of OMVs close to the outer membrane in both capsulated strains, which was in contrast to the close proximity of the OMVs for the unencapsulated mutant, suggesting that the presence of CPS may contribute to the zone of OMV clearing within ∼200 nm of the bacterial outer membrane.

**FIGURE 6:**
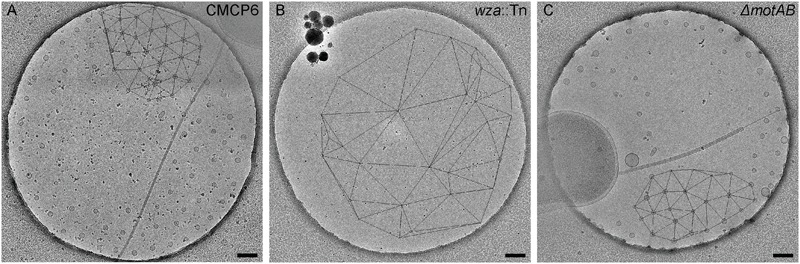
Nearest neighbor distances of *Vibrio vulnificus* OMVs. **(A)** Delaunay triangulation of wild-type CMCP6 OMVs; mean distance is 202 ± 45 nm. **(B)** Delaunay triangulation of *wza*::TnPhoA unencapsulated mutant (FLA1009); mean distance is 471 ± 214 nm. **(C)** Delaunay triangulation of Δ*motAB* flagellar motor mutant (FLA674); mean distance is 212 ± 52 nm. The distribution and mean distance between the wild-type OMVs and the motor mutant OMVs is similar, while the distribution and mean distance for the unencapsulated mutant is larger and more variable. Scale bars, 200 nm.

To assess the nearest neighbor distances between OMVs, we used Delaunay triangulation to calculate the mean center-to-center distances of convex arrangements of OMVs (**Figure 6**). For wild-type CMCP6, the mean distance was 202 ± 45 nm. For unencapsulated mutant the mean distance was 471 ± 214 nm, a much greater variability. And the non-motile mutant had a mean distance of 212 ± 52 nm, similar to wild-type. The diameters of OMVs differ by mutation as well. The mean diameter for wild-type was 47 nm, for the unencapsulated mutant it was 31 nm with greater variability, and 53 nm for the non-motile mutant.

### The Relationship between Cell Motility and OMV Production

To determine if OMV production has any dependence on motility, we performed motility assays of several *V. vulnificus* strains (**Figures 3B,C**). As expected, motility efficiency was drastically reduced when genes encoding motor proteins (Δ*motAB*) or flagellins (Δ*flaCDE*, Δ*flaFBA*) were deleted. Motility was slightly increased when mutations compromised capsule production (**Figure 3B**), which may be attributable to a reduced friction coefficient due to the loss of the polysaccharide capsule. Combining the OMV measurement data with the motilities of individual strains, the non-motile, but still-flagellated mutant produced far fewer OMVs per cell (nearly fourfold fewer). These OMVs remained close to the outer membrane while maintaining a ∼200 nm zone of clearance. Their mean diameters (53 nm) were slightly larger than wild-type (47 nm). To confirm that the OMV phenotypes were the direct result of the mutations, the Δ*motAB* mutation was complemented with a plasmid encoding *motAB* and the Δ*flaCDE*::*cat* mutation of the double Δ*flaFBA*::*aph*, Δ*flaCDE*::*cat* mutant was reverted by allelic exchange via natural transformation. Note that the Δ*flaFBA*::*aph* mutation that remains after *flaCDE* reversion is motile (Gulig et al., 2009). The production and arrangement of the OMVs in the complemented and reverted strains replicated that of wild-type strain CMCP6 (**Supplementary Figure S1**). Because the *wza*::TnPhoA mutation was polar on an extensive operon involved with production of capsule, it was not possible to complement this mutation. Similarly, because there was no selectable marker for reversion, it could not be reverted.

### Arrangement of the Secreted OMVs Is Capsule Dependent

**FIGURE 7:**
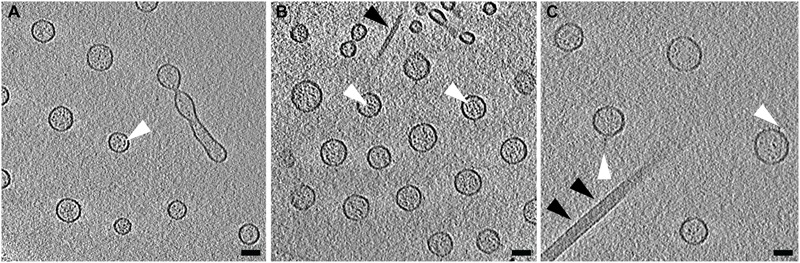
Contents of *Vibrio vulnificus* OMVs. **(A–C)** Averaged central tomographic slices of OMVs. All show electron dense contents inside the vesicle, as well as occasional very small densities on the outside of the membranes, white arrowheads. **(A)** Contains a dividing tube. Single black arrowhead in **(B)** indicates the pilus. Double black arrowheads in **(C)** indicate the flagellum. Scale bars, 50 nm.

Using cryo-ET we are able to reconstruct the bacterium with its surrounding OMVs in 3D. We were able to see that the OMVs were arranged in a plane around the relatively thick cell and did not extend over or under the cell (**Figures 1A,B**). Also evident was the 200-nm region around the outer membrane that contained no OMVs. This particular arrangement of vesicles was abolished in the non-capsulated strain (**Figure 4B**). With respect to OMV content, cryo-ET revealed that cargo packaging was not a requirement for the release of OMVs. Some vesicles appeared empty, while others contained unidentified densities both on the surface and inside (**Figure 7**, white arrowheads).

**FIGURE 8:**
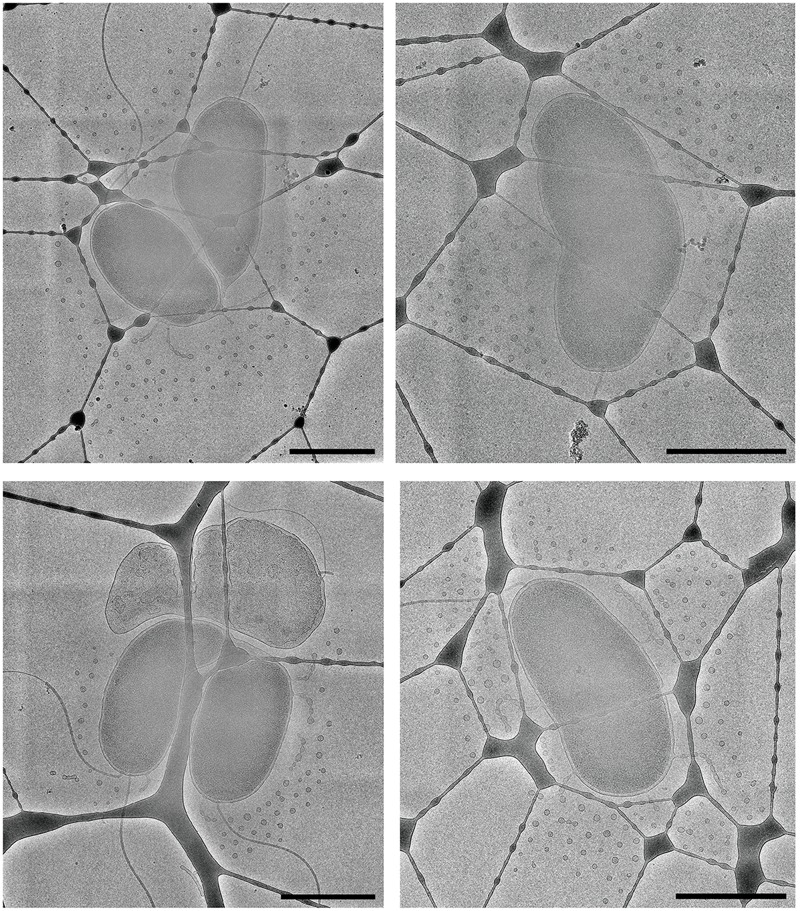
The arrangement of OMVs is not due to the holes in the carbon film support. Wild-type, CMCP6 cells frozen on lacey carbon film supports exhibit a similar OMV arrangement. Scale bars, 1 μm.

The use of Quantifoil holey carbon support films with uniform 2 μm holes raised the question of whether the concentric arrangement of OMVs around the cell was an artifact of the shape of the holes. To assess this, we also imaged vitrified cells on lacey carbon film supports that contain holes of various sizes and shapes. It was determined that the formation of concentric rings by released OMVs was not an artifact caused by the shape of the holes in Quantifoil grids (**Figure 8**). Additionally, nearest neighbor distance calculations for these OMVs were also comparable (∼200 nm) to that of wild-type CMCP6 imaged on Quantifoil grids (**Supplementary Figure S2**).

**FIGURE 9:**
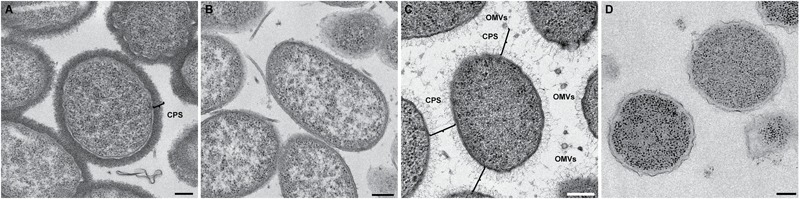
Conventional TEM with heavy metal staining reveals the presence of the capsule. **(A)** Wild-type CMCP6 stained with lysine and ruthenium red prior to resin embedding and thin-sectioning. Note the presence of ∼100 nm thick border of the capsule. **(B)** Unencapsulated mutant *wza*::TnPhoA (FLA1009) stained with lysine and ruthenium red lacks the ∼100 nm capsule. **(C)** Self-pressurized rapid freezing (SPRF) reveals the role of the capsule in OMV distribution. OMVs can be seen evenly spaced between cells. The polysaccharide chains here are approximately 100 nm in length, although this can vary based on biotype. Lines indicate end-to-end interactions between polysaccharide chains that maintain the observed ∼200 nm spacing between cells, between cells and OMVs, and between individual OMVs. **(D)** SPRF of unencapsulated mutant *wza*::TnPhoA (FLA1009) lacks CPS and does not retain OMVs. Scale bars, 200 nm.

Because the bacterial capsule is made of electron transparent polysaccharides and water, it is undetectable by cryo-EM. To visualize the capsule, we first used conventional TEM staining techniques. We used a protocol for lysine-acetate-based ruthenium red-osmium fixation (LRR) to enhance ruthenium red adherence to the capsule. As seen in **Figure 9A**, the capsulated wild-type CMCP6 strain was densely stained with the lysine and ruthenium red (measuring 80–100 nm), while the *wza*::TnPhoA unencapsulated strain in **Figure 9B** was practically devoid of this coating. This method is not quantitative for measurements of capsule thickness due to the potential for specimen dehydration and shrinkage during the staining protocol. Additionally, due to several centrifugation steps in the staining process, the OMVs appear to have been lost, thus providing no information about their arrangement.

To facilitate the preservation of fine structural details such as the outer membrane, the polysaccharide capsule, and the relative positioning of the OMVs, we employed the technique of SPRF, a form of HPF. Bacterial colonies were sealed within a thin copper tube and plunge frozen. The contents of the frozen tubes were freeze-substituted, embedded in resin, and sectioned. In **Figure 9C**, the CPS can be seen extending away from the outer membrane. Their lengths were somewhat variable, but the approximate distances between two cells or between a cell and an OMV were ∼200 nm. This illustrated that the 200 nm zone around *V. vulnificus* cells that is devoid of OMVs in capsulated strains is due to the presence of polysaccharide chains. The unencapsulated *wza*::TnPhoA mutant did not have the polysaccharide layer (**Figure 9D**). Also, as a control, we performed standard HPF of the same wild-type strain, however, the staining of the polysaccharides was less evident (**Supplementary Figure S3**). This phenomenon was due to the cells being packed more densely in the planchettes and for the subsequent processing steps that limited infiltration of stain and substitution media. Nevertheless, there were hair-like projections that were attributed to the polysaccharide capsule.

### Arrangement of OMVs Is Unique to *V. vulnificus*

**FIGURE 10:**
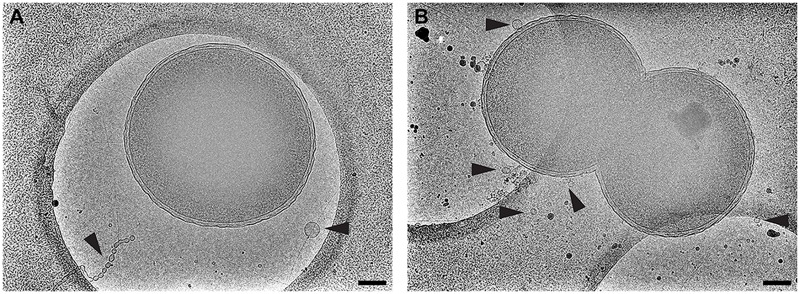
*Neisseria meningitidis* does not form ordered arrays of OMVs. The Gram-negative bacterium *N. meningitidis* is another well-studied OMV producer. Shown is **(A)** serogroup B and **(B)** acapsular mutant m7. Black arrowheads point to OMVs. Scale bars, 200 nm.

We believe that this concentric, planar arrangement of OMVs is exclusive to *V. vulnificus*. We provide a direct comparison to *N. meningitidis* serogroup B (**Figure Figure 10**). Cryo-EM 2D images of *N. meningitidis* cells illustrated that the cells do produce OMVs of variable morphology and dimensions and that the OMVs did not originate from the cells in a periodic fashion or as well-ordered and evenly spaced objects.

## Discussion and Conclusion

Cryo-EM 2D projection images and cryo-ET 3D volumes confirmed previous cryo-EM results (Guerrero-Ferreira et al., 2011), indicating that released OMVs can be found forming a series of planar, concentric rings around the *V. vulnificus* cell (**Figures 1, 2A,B**). The use of mid-log vitrified cells and cryo-EM allowed us to discern this native-state arrangement which would be complicated by other purification and preservation methods (Kulp and Kuehn, 2010). The budding of the 30–60 nm OMVs takes place from the unperturbed, intact outer membranes as either individual vesicles or as segmenting tubes. Only in cells at the late-log growth phase were disruptions of the peptidoglycan layer under the outer membrane seen to contribute to the disordered phenomenon of membrane blebbing (**Figures 2D,F**). We characterized the OMVs, their arrangement and distribution around the cell for wild-type, unencapsulated, and non-motile cells (**Figure 4**). The wild-type strain exhibited the retention of OMVs around the outer membrane, but maintained a zone of clearance of ∼200 nm from the membrane. This was replicated in the non-motile strain, but with fewer OMVs. Complementation or reversion of these strains restored both motility and robust OMV production (**Supplementary Figure S1**). The strain lacking the capsule had many OMVs distributed randomly and over large distances around the cell. Based on our imaging of the polysaccharide capsule by traditional staining (**Figure 9**) as well as HPF techniques (**Supplementary Figure S3**), we hypothesize that the particular spacing between OMVs and between OMVs and the outer membrane is due to the presence of the ∼100 nm thick polysaccharide capsule. Reports that describe other bacteria that produce OMVs, such as *N. meningitidis* (**Figure 10**), *Acinetobacter baumannii* ATCC19606^T^ (Koning et al., 2013), or *Delftia* sp. Cs1-4 (Shetty et al., 2011), show that they do not display clustering associated with the polysaccharide capsule, which demonstrated that this OMV arrangement may be unique to *V. vulnificus*.

The *V. vulnificus* OMVs appeared to have some electron dense content when compared to the periplasmic region of the originating cells and to the vitreous ice background (**Figure 7**). These densities are indicative of their potential role as transportation devices for cellular material, which has been postulated for other bacterial species. It has been established that the OMVs of several strains of bacteria, including *V. vulnificus*, contain toxins, hemolysin, enzymes, outer membrane porins (Ellis and Kuehn, 2010; Kim et al., 2010), and nucleic acids (e.g., RNA) (Sjöström et al., 2015; Koeppen et al., 2016). That OMVs function to transport bacterial RNA to host cells, gives rise to the hypothesis that this is a mechanism for the bacterial pathogen to directly modulate host cell gene expression. It has also been shown that the surface antigen LPS is shed with OMVs as a function of membrane remodeling during stress (Bonnington and Kuehn, 2016) and triggers the caspase-11-dependent cell death pathway upon transport into a host cell’s cytosol (Vanaja et al., 2016). The arrangement of *V. vulnificus* OMVs in a net-like configuration may serve some benefit to the bacterial cell. One function might be to concentrate or localize an immunogenic event to a confined area. In the case of enzyme secretion, concentrated OMVs may benefit the cell by providing localized nutrient liberation and availability.

OMVs have been recognized as important components of bacterial biofilms, however, little is known about their role in the protection and competition of bacterial communities in the environment. Recent studies of the coral pathogen *Vibrio shilonii* AK1 showed that quorum-sensing *N*-acyl homoserine lactones and certain enzymes were packaged within OMVs (Li et al., 2016). The packaging of signaling molecules within OMVs and their subsequent distribution within an ecosystem may provide specific organisms with an evolutionary advantage for colonizing and inhabiting a microbiome. Further comparative studies of OMV release mechanisms, their distribution and the composition of OMVs in Vibrionaceae bacteria, may shed light onto the roles they play on the ability of *Vibrio* species to thrive in a variety of ecological niches.

The unique spatial arrangement of the *V. vulnificus* OMVs suggests that they may be produced in a novel manner. The blebs and tubes and the relationship with CPS reported here could be clues to this process. Similarly, that an organism would evolve to produce OMVs in an unusual manner suggests that these OMVs may serve a unique function in the biology of *V. vulnificus.*

## Author Contributions

CH and EW wrote the manuscript. CH, RG-F, RS, JT, HY, PG, and EW designed and performed the experiments, and edited the manuscript. All authors read and approved the manuscript.

## Conflict of Interest Statement

The authors declare that the research was conducted in the absence of any commercial or financial relationships that could be construed as a potential conflict of interest.

## Supplementary Material

FIGURE S1 Complementation of Δ*motAB*::*aph* and reversion of *flaCDE* in Δ*flaCDE*::*aph* Δ*flaFBA*::*cat*. Strains FLA647 (*motAB*::*aph*), and FLA757 (*flaCDE*::*aph*) are both motile and flagellated. **(A)** Growth in LB media alongside other strains used in the study. The growth curve was performed four times. **(B,C)** Soft-agar motility assay plates at 18 h post-inoculation, spotted in triplicate. The complemented and revertant strains are less motile than CMCP6 or FLA1009. Complemented FLA647 **(D)** and revertant FLA757 **(E)** produce OMVs arranged similarly to motile strains. Scale bars, 200 nm.

FIGURE S2 Nearest neighbor distances of *V. vulnificus* OMVs frozen on lacey carbon film support. Delaunay triangulation of wild-type CMCP6 OMVs; mean distance is ∼230 nm. Scale bar, 200 nm.

FIGURE S3 High pressure freezing confirms the presence of the polysaccharide capsule. Close-packed colonies of wild-type CMCP6 were scraped from agar plates and vitrified in metal planchettes. Frozen cells were then freeze-substituted, resin embedded, and thin-sectioned. Faint densities surrounding the cells are the polysaccharide chains of the CPS. Scale bar, 200 nm.
