# Supplementary figures and images for: The Opportunistic Pathogen Vibrio vulnificus Produces Outer Membrane Vesicles in a Spatially Distinct Manner Related to Capsular Polysaccharide
Source: Front Microbiol. 2017 Nov 7;8:2177. doi: 10.3389/fmicb.2017.02177 (PMC5681939; doi:10.3389/fmicb.2017.02177)

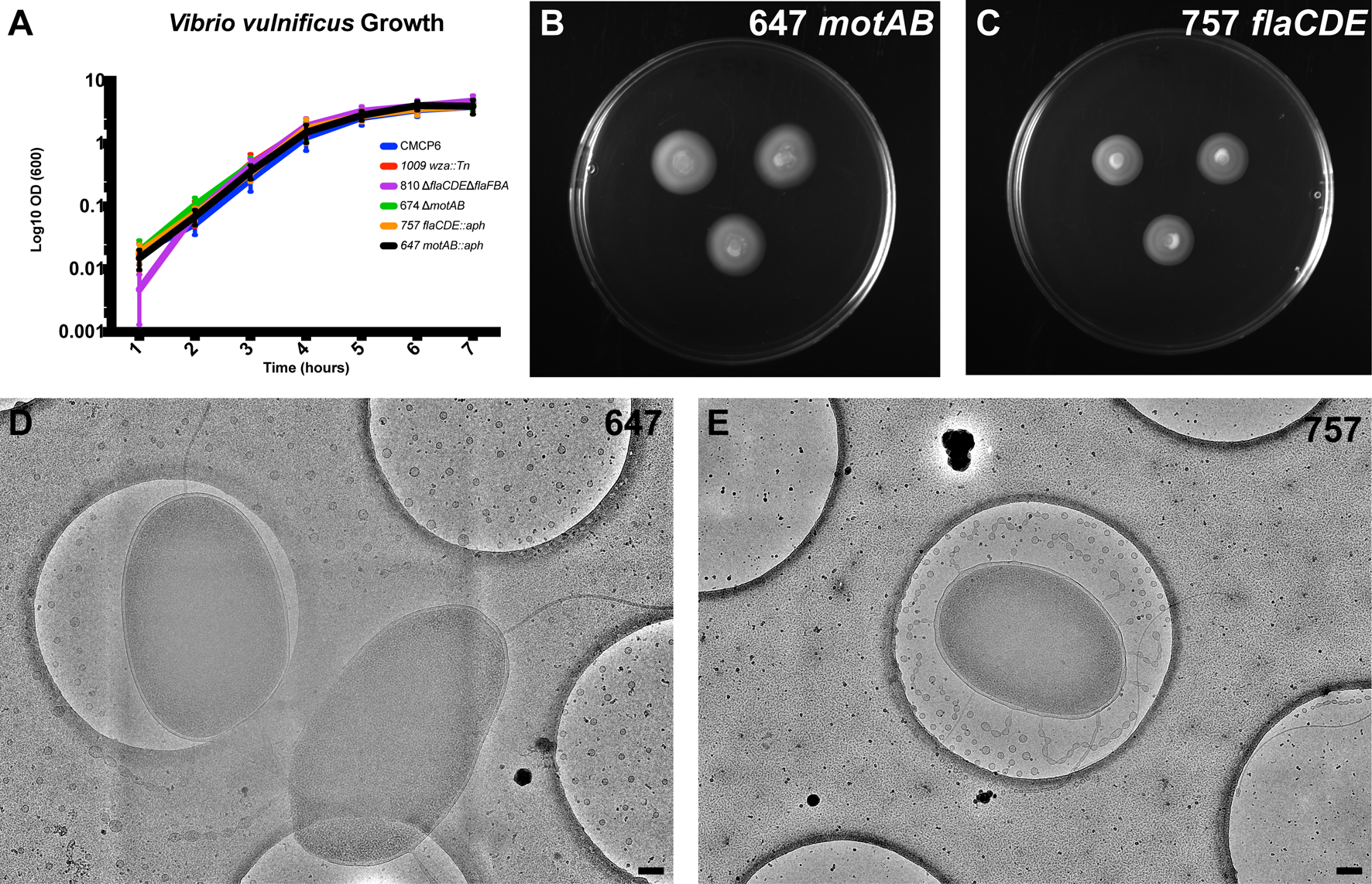

Supplement: FIGURE S1 — Complementation of ΔmotAB::aph and reversion of flaCDE in ΔflaCDE::aph ΔflaFBA::cat. Strains FLA647 (motAB::aph), and FLA757 (flaCDE::aph) are both motile and flagellated. (A) Growth in LB media alongside other strains used in the study. The growth curve was performed four times. (B,C) Soft-agar motility assay plates at 18 h post-inoculation, spotted in triplicate. The complemented and revertant strains are less motile than CMCP6 or FLA1009. Complemented FLA647 (D) and revertant FLA757 (E) produce OMVs arranged similarly to motile strains. Scale bars, 200 nm. [file Image_1.TIF]

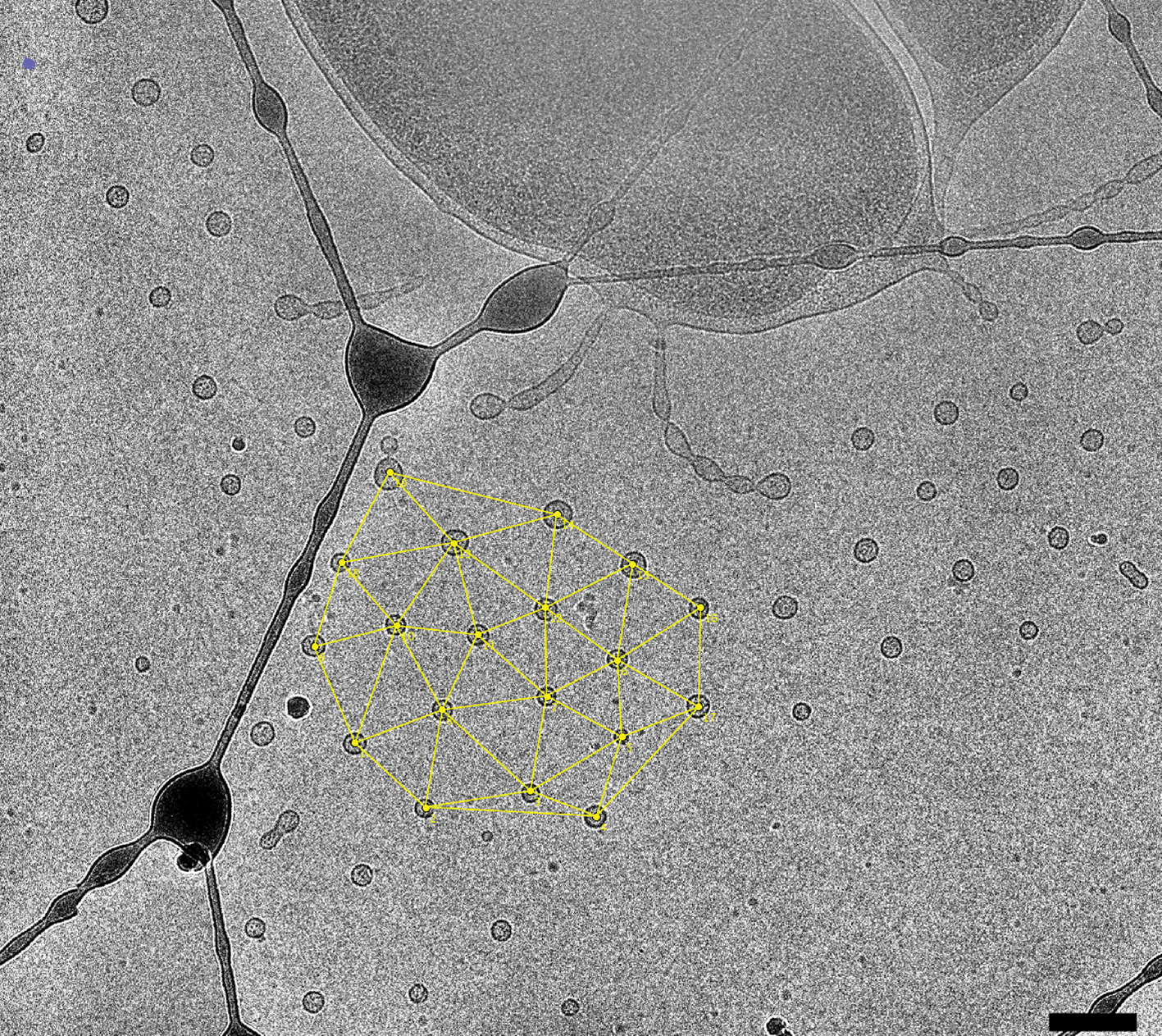

Supplement: FIGURE S2 — Nearest neighbor distances of V. vulnificus OMVs frozen on lacey carbon film support. Delaunay triangulation of wild-type CMCP6 OMVs; mean distance is ∼230 nm. Scale bar, 200 nm. [file Image_2.TIF]

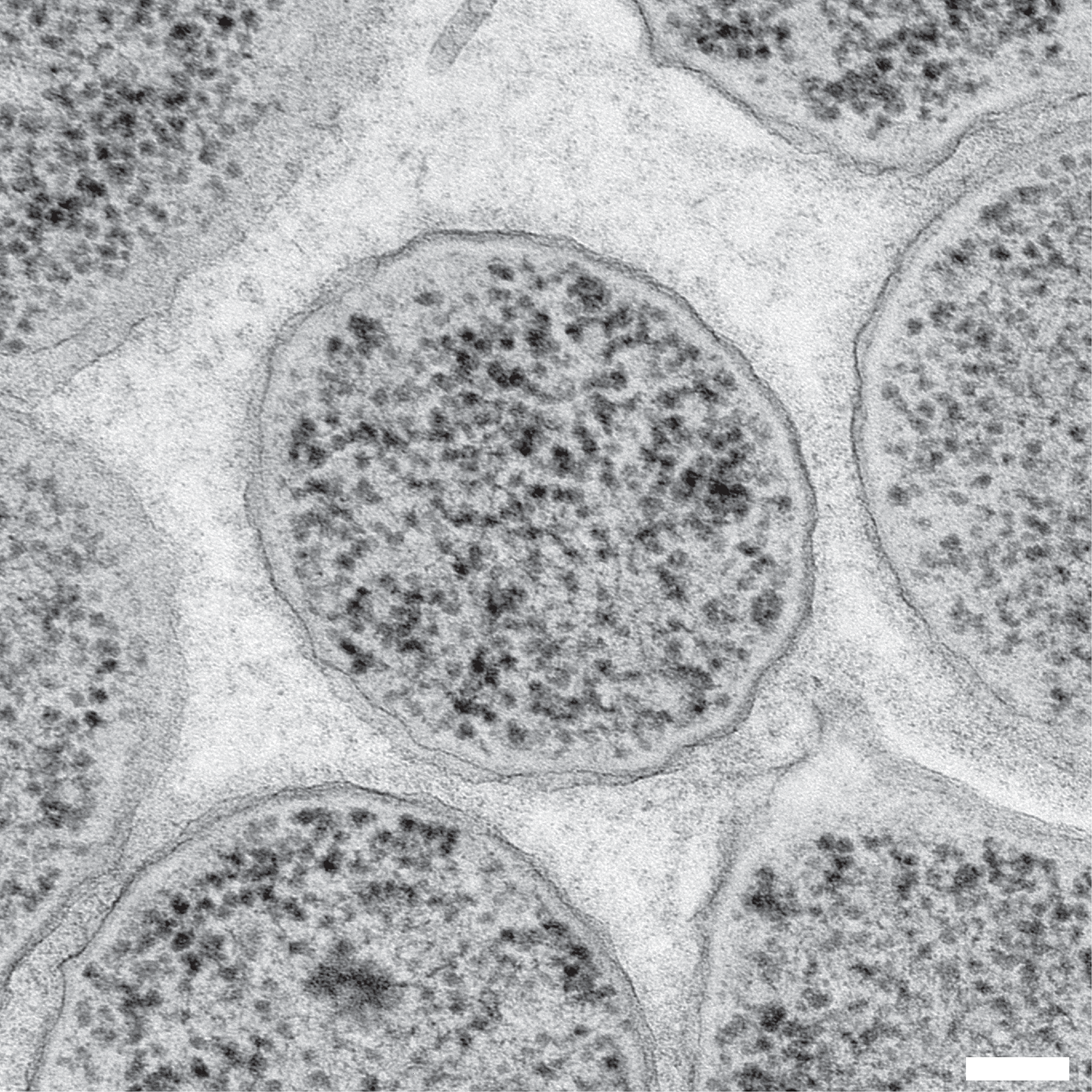

Supplement: FIGURE S3 — High pressure freezing confirms the presence of the polysaccharide capsule. Close-packed colonies of wild-type CMCP6 were scraped from agar plates and vitrified in metal planchettes. Frozen cells were then freeze-substituted, resin embedded, and thin-sectioned. Faint densities surrounding the cells are the polysaccharide chains of the CPS. Scale bar, 200 nm. [file Image_3.TIF]
